# The effect of Pilates exercises on sleep quality and fatigue among female students dormitory residents

**DOI:** 10.1186/s13102-023-00675-7

**Published:** 2023-04-26

**Authors:** Azam Amzajerdi, Maryam Keshavarz, Maryam Ezati, Fatemeh Sarvi

**Affiliations:** 1 School of Nursing and Midwifery, Tehran University of Medical Sciences, Tehran, Iran; 2 School of Nursing and Midwifery, Department of Midwifery and Reproductive Health; Reproductive Sciences and Technology Research Center, Department of Midwifery and Reproductive Health, Iran University of Medical Sciences, Rashid Yasemi St., Valiasr St., Tehran, 1996713883 Iran; 3 School of Nursing and Midwifery, Iran University of Medical Sciences, Tehran, Iran; 4 Department of Public Health, School of Health, Larestan University of Medical Sciences, Fars, Iran

**Keywords:** Pilates exercises, Sleep quality, Fatigue, Dormitory students

## Abstract

**Background:**

This study aims to investigate the effect of Pilates exercises on sleep and fatigue among female college students residing in the dormitory.

**Methods:**

This quasi-experimental study, two parallel groups was performed on 80 single female college students (40 per group), between 18 to 26 years old who lived in the two dormitories. One dormitory was considered as the intervention group and another as the control group. The Pilates group received three one-hour Pilates exercise sessions per week for eight weeks and the control group maintained their routine activities. The Pittsburgh Sleep Quality Index (PSQI) and the Multidimensional Fatigue Inventory (MFI-20) were used respectively to assess sleep quality and fatigue levels, at three time points: baseline, end of week four, and eight follow-ups. Fisher’s exact, Chi-square, independent sample t-test and repeated measurements were used.

**Results:**

Overall, 66 participants completed the study (32 and 35 participants in the Pilates and control groups, respectively). After four and eight weeks of intervention, the overall mean score of sleep quality improved significantly (*p* < 0.001). At week four of the intervention, the Pilates group had a significantly lower mean score for subjective sleep quality and daytime dysfunction than the control group (*p* < 0.001 and *p* < 0.002, respectively), although sleep duration and habitual sleep efficiency improved after eight weeks of intervention (*p* < 0.04 and *p* < 0.034, respectively). Additionally, the overall mean score of fatigue and its dimensions in weeks four and eight of the intervention in the Pilates group were significantly lower compared to the control group (*p* < 0.001).

**Conclusion:**

After eight weeks of Pilates exercises implementation, most components of sleep quality significantly improved; however, the effect of Pilates exercises on fatigue was evident from week four onward.

*Trial registration* This trial was registered on 2/6/2015 in the Iranian Registry of Clinical Trials with the IRCT ID: IRCT201412282324N15**.** URL of registry: https://www.irct.ir/trial/1970.

## Background

Sleep is a complex physiological and behavioral process that is important for physical and mental health [1]. It is helpful for many supporting functions of life [2]. Poor sleep quality is a severe public health problem [3], and prevalent among the general university population [4]. Poor sleep quality decreases health quality and is associated with physical and psychological problems [5]. Sleep quality and quantity may influence people’s performance [6] and have been documented as a challenge for college students [7]. According to a China’s education ministry report, approximately 31.43 million students live in residence halls [8]. According to another account, between 16 and 23% of young adults exhibit symptoms of poor sleep quality [4]. Based on a recent study, 75% of surveyed college students reported having sleep problems such as delayed sleep phase syndrome, difficulty falling asleep, sleep disturbances, and excessive daytime sleepiness [9]. Studies show that students go to bed at varying times, do not get enough sleep, have poor sleep quality, and use stimuli to stay awake [10]. Insufficient sleep has been shown to affect academic performance [1, 7] and is associated with anxiety, depression, and an increased risk for other psychiatric disorders [11]. Female college students, as per reports, have a longer sleep latency, a higher rate of nocturnal awakenings, and a lower quality of sleep than male college students [9].

Sleep is curative and alleviates fatigue, enabling individuals to face a new day’s challenges [12]. Fatigue is one of the most common complaints when seeking medical advice in primary care [5], known to cause disability, burnout, yawning, drowsiness, and lack of interaction [13]. A study reported that 19.8% and 67.2% of Iranian university students suffered from severe and moderate fatigue, respectively [14]. Sleep quality has been linked to the severity of fatigue in studies, and the severity of fatigue increases as sleep quality deteriorates [12].

Academic sleep associations recommend physical exercise as a low-cost, easily administered, non-pharmacologic intervention to improve sleep [11]. Pilates exercises are a kind of exercise that concludes aerobic, resistance and strength components [15]. Pilates-based exercises have been widely used in healthy subjects due to their beneficial effects [3]. Various studies have demonstrated the positive effects of Pilates exercises on the quality of sleep [7, 16–18] and fatigue [7, 19–23] using different activity designs and populations. A meta-analysis of six randomized trials involving 477 participants concluded that all studies reported a beneficial effect of Pilates exercises on the total score of sleep quality [3]. Yet in scoping the literature, there are seemingly few studies exploring the effect of Pilates exercises on sleep quality and fatigue in college students. In one study Pilates exercises improved sleep quality in 41 college-aged individuals, although the effect of the intervention on the components of sleep quality has not been reported [24]. One study also showed that the combined movements of Pilates and Yoga over a long period of 12 weeks had a positive effect on skeletal muscle mass, basal metabolism, and body satisfaction in female college students [25]. These findings indicate a need to explore the efficacy of evidence-based interventions.

Living in a student dormitory could impact sleep quality among students [26]. Because health professions’ curricula, such as medicine, nursing, and pharmacy, are more intense and tiring, these students experience sleep problems more frequently [10]. In a study of 658 students at one university of medical sciences in Iran, 49.2% had poor sleep quality [14]. Our study’s main objective was to examine the effects of Pilates exercises on sleep quality and fatigue in medical sciences students. We hypothesized that participants who complete an eight-week Pilates exercise program will experience significant improvements in sleep quality and decreased fatigue.

## Methods

### Study design

This was a quasi-experimental study with two parallel groups of students living in two Iran University of Medical Sciences (IUMS) dormitories, in Tehran, Iran. Sampling was done from October to December 2015. The name of eight dormitories were written on separate cards and then were placed in opaque envelopes. Two envelopes were randomly selected by a colleague. The first selection of dormitory was considered for the intervention group and the second for the control group. Definitely the welfare conditions are similar in all dormitories of IUMS. In both of two dormitory students’ meals were prepared by self-service of university. The groups were similar in terms of food intake, also, the intake of micronutrients and side dishes.

### Participants

This study enrolled female college students between 18 to 26 years old. Participants were excluded if they had any one of the following: BMI > 29 kg/m^2^, smoking, sleeping medications, complementary medicine use over the last six months, stressful events in the past three months, presence of a physical or mental illness, or surgical history. Participants were also excluded during the intervention period included absence for three consecutive or five non-consecutive exercise sessions, any regular physical activity other than the intervention of study, using complementary medicine or herbal therapy that affects sleep or fatigue during the intervention period (i.e., energy-enhancing drugs, daily consumption of caffeinated drinks and chocolate or sleeping medications).

### Sample size

Sample size was obtained as 32 participants in each group based on related study [24].The power was set at 80%, with a 5% significance level. However, considering a 20% as dropout rate, we recruited 40 students per group. The effect size was assumed to be 1.

### Study instruments

The Pittsburgh Sleep Quality Index (PSQI) measures subjective sleep quality, sleep latency, sleep duration, sleep efficiency, sleep disturbances, use of sleeping medications, and daytime dysfunction [27]. Each item is rated on a Likert scale ranging from 0 to 3, giving a total score of 0 to 21. A higher score indicates a lower sleep quality [28]. We excluded students taking sleeping medications from this study, which resulted in all students receiving a score of zero for area six (the use of sleeping medications). In one study the psychometric properties of the PSQI have been evaluated and the reliability of the Persian version of PSQI as assessed by Cronbach’s alpha coefficient was reported to be 0.77 [29]. In the current study, Cronbach’s alpha coefficient for the PSQI was 0.80.

The Multidimensional Fatigue Inventory (MFI-20) is a 20-item questionnaire that assesses five dimensions of fatigue: general fatigue, physical fatigue, mental fatigue, decreased activity, and decreased motivation [30]. Each part has a score ranging from 4 to 20. The total score for fatigue level is the sum of the scores for each of the five components (i.e., the total score ranges from 20 to 100). Higher scores indicate severe fatigue. The validity and reliability of the MFI-20 have been previously established [31]. The reliability of the Persian version of MFI-20 was well reported elsewhere [32]. In the current study, Cronbach’s alpha coefficient for the MFI-20 was 0.85.

The procedure was explained to the participants who met the inclusion criteria. All participants signed informed written consent and completed the demographic, PSQI, and MFI-20 questionnaires. In three one-hour weekly sessions, the experimental group implemented Pilates exercises with a specialized coach for eight consecutive weeks. Each session began with preparation, followed by Pilates activity and relaxation. The first 15 min of each session were spent warming up, followed by 30 min of strength and stretching movements (first standing, then sitting), and finally, 15 min were spent cooling the body. The coach fully supervised how the participants implement the Pilates exercise. The schedule was from 5:00 to 6:00 PM. The primary researcher supervised the regular implementation of Pilates exercises. All participants completed the PSQI and MFI-20 questionnaires during the fourth and eighth weeks of the intervention. All questionnaires were gathered by a research assistant who was blind to the aims of the research. Students in the control group performed their typical daily routines.

### Statistics analysis

The analysis was conducted using SPSS version 25. The normal distribution of quantitative data was evaluated using the Kolmogorov–Smirnov test. The independent t-test was employed to compare quantitative variables between the two groups. The Fisher’s exact and Chi-square tests were also used to compare qualitative data. Furthermore, repeated measurements were used to compare sleep quality and fatigue levels between the two groups over time. The Greenhouse–Geisser test was also used in cases where the sphericity of the test was not assumed. A result of < 0.05 was considered significant in all tests.

## Results

**Fig. 1:**
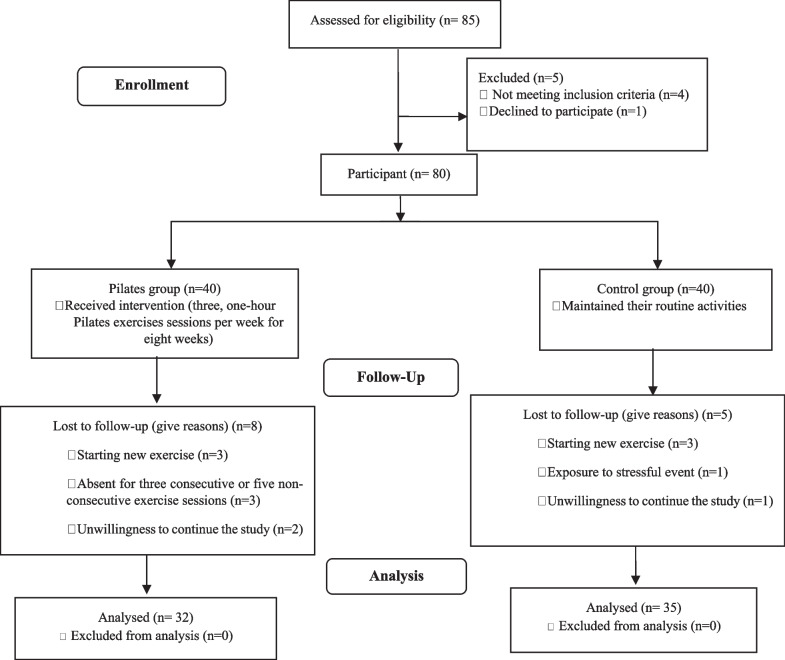
Consort diagram

Eighty participants were recruited to this study (40 students per group). In total, 67 participants (32 and 35 participants in the Pilates and control groups, respectively) completed the study. Excluded cases were six students due to starting a new exercise (three participants in each group) and three participants due to unwillingness to continue the study (two and one participant in the Pilates and control groups, respectively). A further three participants were removed due to irregular participation in sessions training in the Pilates group and one participant due to exposure to a stressful event in the control group (Fig. 1).

There were no significant differences observed between the two groups regarding socio-demographic data (Table 1). The baseline sleep quality score of the participants was similar. After four weeks of intervention, in the Pilates group significantly improved the total mean score of sleep quality and two components (subjective sleep quality and daytime dysfunction) compared to the control group. After eight weeks of intervention, in the Pilates group, the total mean score of sleep quality and its components (except for sleep latency and sleep disturbance) was significantly lower than that of the control group (Table 2).

**Table 1:** The characteristics of the study samples^a^

Variables	Pilates group (n = 32)	Control group (n = 35)	P-value
Age (years)	20.75 ± 1.27	20.08 ± 1.31	0.1^b^
BMI (kg/m^2^)	22.13 ± 2.01	22.78 ± 2.34	0.17^b^
Occupational status			
Employed	5 (15.6)	8 (22. 9)	0.45^c^
Unemployed	27 (84.4)	27 (77.1)	
Household income			
Good	32 (100)	32 (91.4)	0.24^d^
Intermediate	0 (0)	3 (8.6)	
Field of Study			
Nursing and midwifery	7 (21.90)	12 (34.3)	0.33^c^
Management and statistics	12 (36.45)	13 (37.14)	
Nutrition and health	8 (25.33)	6 (17.14)	
Others	5 (16.32)	4 (11.42)	

^a^Values are expressed as NO (%) or Mean ± SD; BMI: Body Mass Index

^b^Independent sample t-test

^**c**^Chi-square test

^d^Fisher’s exact test

**Table 2:** Comparison of sleep quality between two groups at 4 and 8 weeks of intervention

Variables	Groups		Baseline			Four weeks follow-up			Eight weeks follow-up		
			Mean ± SD^♣^	*p*-value^a^		Mean ± SD^♣^		*p*-value^a^	Mean ± SD^♣^	*p*-value^a^	*p*-value^b^
Subjective sleep quality	Pilates	0.90 ± 0.53		0.3		0.25 ± 0.43		< 0.001	0.09 ± 0.29	< 0.001	< 0.001
	Control	0.77 ± 0.64				1.14 ± 0.64			1.40 ± 0.65		
Sleep duration	Pilates	1.21 ± 0.83		0.54		0.96 ± 0.75		0.3	0.75 ± 0.43	< 0.04	0.229
	Control	1.05 ± 0.63				1.20 ± 0.93			1.20 ± 0.93		
Sleep latency	Pilates	0.56 ± 0.71		0.92		0.62 ± .0.70		0.7	0.78 ± 0.55	0.474	0.995
	Control	0.54 ± 0.61				0.71 ± 0.78			0.71 ± 0.78		
Habitual sleep efficiency	Pilates	1.40 ± 1.52		0.28		0.53 ± 0.67		0.06	0.15 ± 0.36	0.034	< 0.012
	Control	1.80 ± 1.49				1.31 ± 1.43			0.48 ± 0.70		
Sleep disturbance	Pilates	1.09 ± 0.39		0.75		1 ± 0.35		0.77	0.93 ± 0.35	0.980	< 0.777
	Control	1.05 ± 0.48				0.97 ± 0.45			0.94 ± 0.48		
Daytime dysfunction	Pilates	1.12 ± 0.83		0.11		0.59 ± 0.71		< 0.002	0.34 ± 0.48	< 0.0001	0.003
	Control	0.82 ± 0.51				1.20 ± 0.79			1.14 ± 0.73		
Global PSQI score	Pilates	6.34 ± 2.70		0.81		5.40 ± 2.28		< 0.001	5.03 ± 2.11	< 0.0001	< 0.002
	Control	6.31 ± 2.62			7.62 ± 2.51				7.57 ± 2.70		

^♣^Mean ± standard deviation, expected range for each variable: (0–3; 0 = best result; 3 = worst result)

^a^Independent sample t-test

^b^Greenhouse-Geisser test

At baseline, the two groups had comparable fatigue levels. At four and eight weeks following the Pilates exercises, the total mean score of fatigue and its dimensions were significantly lower in the Pilates group than those of the control group, indicating that the Pilates group experienced a decrease in fatigue levels and its components (Table 3).

**Table 3:** Comparison of fatigue level between two groups at 4 and 8 weeks of intervention

Variables	Groups	Baseline		Four weeks follow-up		Eight weeks follow-up		
		Mean ± SD	p-value^a^	Mean ± SD	p-value^a^	Mean ± SD	*p*-value^a^	*p*-value^b^
General fatigue	Pilates	12.71 ± 1.48	0.49	10.18 ± 1.55	< 0.001	7.81 ± 2.17	< 0.001	< 0.001
	Control	12.54 ± 0.61		12.14 ± 1.19		12.17 ± 0.74		
Physical fatigue	Pilates	12.31 ± 1.55	0.66	10 ± 1.54	< 0.001	7.21 ± 1.79	< 0.001	< 0.001
	Control	12.37 ± 0.73		15.97 ± 1.11		12.08 ± 0.70		
Mental fatigue	Pilates	12.21 ± 1.40	0.37	9.75 ± 1.48	< 0.001	7.03 ± 1.67	< 0.001	< 0.001
	Control	12.17 ± 0.74		11.82 ± 1.20		11.74 ± 0.74		
Reduced activity	Pilates	12.18 ± 1.40	0.17	9.50 ± 1.58	< 0.001	6.93 ± 1.70	< 0.001	< 0.001
	Control	11.94 ± 0.63		11.60 ± 1.21		11.54 ± 0.70		
Reduced motivation	Pilates	12.06 ± 1.50	0.49	9.37 ± 1.56	< 0.001	6.81 ± 1.78	< 0.001	< 0.001
	Control	11.80 ± 0.58		11.37 ± 1.16		11.54 ± 0.70		
Global MFI score	Pilates	61.50 ± 7.14	0.27	48.81 ± 7.57	< 0.001	35.75 ± 9.02	< 0.001	< 0.001
	Control	60.85 ± 2.86		48.40 ± 7.77		58.91 ± 3.35		

^a^Independent sample t-test

^b^Greenhouse-Geisser test

The Greenhouse–Geisser test revealed an improvement in the mean score of sleep quality and its components during the time in the Pilates group (except for sleep duration, sleep latency, and sleep disturbance) (Table 2). Overall, the mean total score of fatigue level and its components significantly decreased in the Pilates group over time (Table 3).

## Discussion

The purpose of this study was to determine the effect of an eight-week Pilates exercises on sleep quality and fatigue levels in female college students who resided in dormitories. After four weeks, findings indicated that sleep quality and two components had improved (subjective sleep quality and daytime dysfunction). Additionally, eight weeks of intervention improved sleep duration, habitual sleep efficiency, and daytime dysfunction. Notably, increasing the duration of Pilates exercises resulted in significant improvements in the majority of sleep quality components. The study’s findings showed that, four and eight weeks of Pilates exercises significantly improved fatigue levels and its components, indicating that even four weeks of Pilates exercises could be enough to reduce fatigue. According to repeated measurements, eight weeks of Pilates exercises improved both the total mean score of sleep quality and its component (except sleep duration, sleep latency, and sleep disturbance), as well as a decrease in the total mean score of fatigue levels and its components has also been shown at different points in time.

Various studies have reported the positive effects of Pilates exercises on sleep quality in different populations. In one study, a 12-week Pilates exercise program significantly improved sleep quality in a sedentary young population [16]. Another study found that after eight weeks of Pilates exercises, subjective sleep quality, daytime dysfunction, and the global PSQI score were improved in primigravida postpartum women [17]. One study on 110 postmenopausal women reported improvement in sleep quality in all domains after 12 weeks of Pilates exercises [5]. In another study, 16 weeks of Pilates exercises improved the PSQI total score, sleep latency, and use of sleeping medications in older women [18]. In the case of Pilates exercises, randomized clinical trials examining their effects on sleep quality and fatigue in college students are scarce. In one study, after fifteen weeks of Pilates exercises, college students reported improvement in sleep quality, but there was no report about sleep components [24]. Another study evaluated the effect of combined program exercise (Pilates and Yoga movements) conducted on 21 female college students. This study confirmed that the combined treatment exercise program is effective to form a correct body image, positively affecting the muscle mass and basal metabolism of students [25].

In College, students acquire critical knowledge, skill, human capital, and credentials that will enable them to find successful employment and contribute to society following graduation [8]. Epidemiologic studies identify college students as a population at risk for insufficient sleep [1]; additionally, fatigue through the performance of activities and vital life roles has a significant impact on many aspects of people’s lives [14]. The current study revealed that four and eight weeks of Pilates exercises significantly improved fatigue levels and its components in college students. Our results are consistent with studies that present the Pilates method as an alternative and effective method to improve fatigue in postpartum women [20], postpartum depressed women [19], people with Multiple Sclerosis [21, 22], Multiple Sclerosis with minimal-to-mild mobility disability [23] and postmenopausal women [5]. In a parallel study, following a four-week intervention, aerobic exercise significantly decreased the total fatigue score and its components [33].

Sleep quality is an important predictor of physical and mental health. Poor sleep quality may lead to fatigue, drowsiness and mood swings and improper physical performance [34]. Exercise as a non-pharmacological intervention can indirectly improve mental health by enhancing sleep quality [35]. Activity constitutes a therapeutic behavior that can improve body composition and fitness, which is critical in enhancing sleep quality [3]. In an intervention conducted parallel with the current study, eight weeks of aerobic exercise positively affected all sleep quality components compared to the control group. Considering that the participants were experiencing exercises for the first time, the design of the study was such that the intensity of exercises ranged from mild at the beginning and then to severe [33]. The positive effect of the intervention could be attributed to the design of the intervention, which undoubtedly, was not possible in the current study. A systematic review and meta-analysis of five randomized clinical trials (n = 660 women, mean age 48.6–55.8 years) reported that 12–16 weeks of moderate physical activity (aerobic exercise excluding yoga) improved sleep quality [36]. The results of a meta-analysis included 32 randomized clinical trials showed that exercise training is effective in improving sleep quality of participants with sleep disturbance in different age range [35]. A meta-analysis of six randomized trials corroborates Pilates exercises beneficial effects on sleep quality in people in different age range. In contrast, there was no significant improvement in the use of sleep medications [3]. Because none of the participants in our study used sleeping medications, they received a zero for PSQI area six (the use of sleeping medications), removed from the list of sleep quality questionnaires.

It was demonstrated that low physical activity was significantly negatively associated with poor sleep [37]. It has been suggested that increased serotonin levels in the brain due to exercise can influence the circadian rhythm, thereby improving sleep quality [16]. Pilates activity is mind–body training that focuses on improving strength, core stability, flexibility, muscle control, balance, and breathing [38]. Due to Pilates activity, sympathetic nervous system activity may decrease, while parasympathetic nervous system activity may increase. These physiological changes have caused many positive physical and mental effects [39].

Pilates has become a form of exercise to improve physical and mental health [40], and also is considered an attractive mainstream exercise especially in women worldwide [41]. It is an increasingly popular exercise modality that is reported to exert beneficial physiological effects on the body [42]. Due to the high rate of inactivity and lack of access to sports facilities among female dormitory students, Pilates exercises as a low-cost activity can be a practical step toward improving the quality of sleep and health of this group of young people. The study’s inability to control participants’ daily sleep hours and routines can be considered one of its limitations.

## Conclusions

Eight weeks of Pilates exercises is evidenced to improve students’ fatigue and most components of sleep quality in female college students resident in the dormitory. By increasing the duration of the Pilates exercises, the effects of activity on sleep quality may be more likely to emerge. Future studies with longer interventions are needed to examine the effect of Pilates exercises on all components of sleep quality.

## Data Availability

If necessary, the data in the current study are available upon reasonable request from the corresponding author.

## Abbreviations

BMI: Body mass index
MFI-20: Multidimensional fatigue inventory
PSQI: Pittsburgh sleep quality index
